# HIV transmission by human bite: a case report and review of the literature—implications for post-exposure prophylaxis

**DOI:** 10.1007/s15010-020-01477-6

**Published:** 2020-07-26

**Authors:** Dirk Schürmann, Christian Hoffmann, Miriam S. Stegemann, Christoph Ruwwe-Glösenkamp, Lutz Gürtler

**Affiliations:** 1 Department of Infectious Diseases and Pulmonary Medicine, Charité-Universitätsmedizin Berlin, Berlin, Germany; 2 Infektionsmedizinisches Centrum Hamburg, ICH Stadtmitte, Glockengiesserwall 1, 20095 Hamburg, Germany; 3 Max von Pettenkofer-Institut für Hygiene und Medizinische Mikrobiologie, Ludwig-Maximilians-Universität, Munich, Germany

**Keywords:** Human immunodeficiency virus, HIV transmission, Route of transmission, Human bite, Post-exposure prophylaxis

## Abstract

We report a case of a probable HIV-1 transmission by human bite. The analyzed data from ten previously reported  suspected or allegedly confirmed HIV transmissions revealed a deep bleeding bite wound as the primary risk factor. A high HIV plasma viral load and bleeding oral lesions are present most of the time during HIV transmission by bite. HIV post-exposure prophylaxis (PEP) should be recommended in case of a bleeding wound resulting from a bite of an HIV-infected person. PEP was missed in this presented case.

## Introduction

Counselling about post-exposure prophylaxis (PEP) for prevention of HIV infection following potential exposure after a human bite is an infrequent but regular task for infectious disease specialists. The spectrum of potential bite victims includes private persons in a quarrel, persons incarcerated in correction facilities, and healthcare staff being bitten by patients during aggressive encounters or while handling seizures [1].

PEP counselling on HIV transmission by human bite has been a regular consultation request in our infectious disease center, reaching an estimated number of up to ten cases per year. So far, recommendations for HIV-PEP have been inconsistent. While some consider the risk of HIV transmission by human bite to be negligible and do not advocate PEP, others see an actual risk and do recommend PEP.

A recent review about the risk of HIV transmission by human bite concluded that the risk appears to be negligible [2]. The number of assumed HIV transmissions was small, while a large number of patients exposed to bites of HIV-infected patients did not seroconvert.

We report a case of probable HIV-1 transmission by human bite. Additionally, we analyzed available data from suspected or allegedly confirmed HIV transmissions. The goal was to assess the plausibility of claimed HIV transmission by bite, to reveal conditions potentially leading to HIV transmission, and to define the indication for PEP in such instances.

## Case report

A 42-year-old never married man was bitten in his left hand in 2004 by a friend during an argument. Details are summarized in Table 1, case 11. The bite was cared for and documented by an employee of a surgical outpatient clinic. There was a gaping bleeding wound which was not sutured. Although the patient acknowledged the presence of an HIV infection of the aggressor, no PEP was recommended. The HIV–ELISA–screening test performed by a general practitioner 7 days after the incident was negative. Ninety days later (97 days after the bite incident), a HIV–ELISA antibody–screening test was positive and confirmed by Western blot. However, the patient did not show up again because of depression and alcohol problems. Nineteen months after the bite incident, he presented at Charité, the University Hospital of Berlin with thoracical herpes zoster. HIV-1 infection was confirmed by Western blot (CD4 cell count 415/µl, viral load 132,000 HIV RNA copies/ml). He imperturbably denied any further potential risk factors for acquiring HIV during the past 12 months prior to the bite until 97 days after the bite when the first test turned out to be positive. He did not remember any signs and symptoms of acute retroviral syndrome.

**Table 1:** Case reports of suspected or allegedly confirmed HIV transmission by human bite

Case	Authors, year of publication (reference)	Bite victim				Circumstances of bite	Aggressor			HIV-type relatedness (victim/aggressor) assessed by authors	Authors´ assessment of causality of bite and transmission
		Sex, age (years), history	Bite location	Wound features	Seroconversion or acute HIV infection		Relationship with victim, sex, age (years), risks	HIV status at the time of bite	CD4 cells/ml blood, VL: HIV RNA copies/ml		
1	Wahn et al. (1986) [6]	Male, 8	Forearm	Teeth imprints, no bleeding or haematoma	Not reported	Possibly seizure	Brother, 5	AIDS	Not reported	Not evaluated	Bite likely route
2	Anonymous (1987) [7]	Female, 26	Leg	Not reported	Seronegative ~ 1.5 years before bite, seropositive ~ 2 years after bite	Fight, leading to a teeth loss of aggressor and bleeding mouth	Sister, IV drug abuse	HIV infection known	Not reported	Not evaluated	Bite most likely route
3	Vidmar et al. (1996) [8]	Male, 53	Left index-finger nail	Small crack and shallow wound, teeth imprints, no visible bleeding	Acute infection, seroconversion 54 days after bite	Seizure, bite wound on aggressor´s tongue and blood in saliva	Neighbour, male, 47	AIDS	High level of HIV copies in plasma 11 months earlier, at the time of bite on zidovudine treatment	Not evaluated	Case shows that HIV transmission is possible by bite
4	Anonymous (1996) [9] Liberti et al. (1996) [10]	Male, 91	Hand, arm, leg	Severe tissue damage	Seroconversion within 6 weeks after bite	Fight, dental problems, bleeding gums	Robber, female prostitute	HIV infection known	Not reported	Confirmed ^a^	Evidence support HIV transmission by bite
5	Khajotia et al. (1997) [11]	Male, 20	Lip	Cut inside of the lower lip with taste of blood	Seroconversion following negative tests over 7 months after bite	Deep kiss during a night club visit	Call girl, one time contact	HIV status unknown	Not reported	Not evaluated	Male virgin contracted HIV by lip bite
6	Andreo et al. (2004) [12]	Female, 59, widow	Hand	Deep wound needing suture	Acute infection, seroconversion 40 days after bite	Seizure, blood in the mouth	Son, 31	AIDS	Not reported	Confirmed ^b^	Unequivocal evidence
7	Bartholome et al. (2006) [13]	Female, 3	Middle finger of left hand	Bleeding at the site of bite	Seropositive ~ 4 years after bite	Bite reason not reported, dental caries, bleeding gums	Father	HIV status unknown, AIDS 3 years after bite	Not reported	Not evaluated	HIV infection believed to be a direct result from bite
8	Uzoigwe et al. (2007) [14]; Akani et al. (2007) [15]	Female, 30, pregnant	Upper lip	Bleeding injury necessitating suturing	Seronegative shortly prior to bite, seropositive ~ 16 months after bite	Fight	Sister in law, commercial sex worker	HIV infection known, no symptoms	Not reported	Not evaluated	HIV infection is associated with the bite
9	Deshpande et al. (2011) [16]; Jadhav 2018 [17]	Married man 44, diabetes mellitus	Left thumb	Nail lost with raw bleeding nail bed	Acute infection, VL 2,470,000 copies/ml 28 days after bite	Argument, good oral hygiene, no bleeding oral lesions	Foster son	HIV infection known, no symptoms	CD4 cells: 383, VL plasma: 17,163, VL saliva: 2,405 VL salivary cells: 161	Confirmed^c^	Possible
10	Thomas et al. (2019) [18]	Male, 56	Left cheek and thumb	Deep injuries, thumb fracture	Acute infection, VL 1,023,292 ( 6.01 log_10_) copies/ml 41 days after bite	Fight, blood-stained saliva from bite injuries	Not reported, 34	HIV infection known	VL 200 (2.3 log_10_) 29 days after the bite while on ART since 6 weeks prior to the bite	Confirmed^d^	Confirmed
11	Schürmann et al. (2020), case report of this article	Unmarried man, 42, alcohol use	Ball of left hand	Bleeding gapping wound	Seronegative 7 days after bite, seropositive 97 days after bite	Argument	Acquaintance, male, 23, IV drug use	HIV infection known, no symptoms	CD4 cells: 243, VL plasma: 176,000 5 weeks prior to bite	Probable^e^	Probable

*ART* antiretroviral therapy, *IV* intravenous, *VL* viral load. Cases listed in the chronological order which they were reported. Data listed where available. Further information displayed in the text

^a^DNA sequencing showed a close genetic relationship of HIV-1 strains from bite victim and aggressor (no detailed data given), consistent with person-to-person transmission; ^b^both viruses belonging to HIV-1 clade B, same quasispecies of bite victim and aggressor; ^c^91% homology of the C2-V3 region of gp120 , HIV-1C env gene with 5 NLG sites in bite victim and 6 NLG sites in aggressor, presence of single NLG site at V3 region of HIV-1C env gene in bite victim and aggressor; ^d^HIV-1 comparison showed 99% homology of the reverse transcriptase and protease amino acids. ^e^On the basis of an usual mutation rate, the time of HIV-1 transmission or strain identity of aggressor and bite victim, respectively, was calculated to be 42 months prior to blood drawal for virus comparison. The factual time between assumed virus transmission by bite and blood drawal was 27 months

The bite victim and the aggressor were interviewed about the circumstances of the bite incident. Both denied having had any sexual activities with each other or having shared any devices bearing the risk of potential HIV transmission and agreed to perform comparative virus nucleic acid sequencing. The sequences of the viruses showed close relatedness. Both viruses belonged to HIV-1 group M subtype B. Based on the usual mutation rate, the date when the viruses might have had a common ancestor virus was calculated. Considering a usual mutation rate of 1–3 × 10^−3^ nucleotide substitutions per site per year [3, 4], both strains were calculated to have had a common ancestor virus about 42 months prior to the blood withdrawal. The bite occurred 27 months prior to blood examination. Of note, the aggressor suffered from an advanced immunodeficiency with high viral load (Table 1, case 11) which can be associated with a higher mutation rate [5]. Based on the inherent variability of the mutation rate, we considered that the calculated date fitted well into the factual time of the bite event.

## Analysis of cases with HIV transmission by human bite

A comprehensive literature research was conducted in PubMed database up to March 31st, 2020 using the following search terms: “HIV transmission by human bite”, and “HIV transmission” combined with “human bite”. In addition, we screened the cited literature of cases reported and journal review articles dealing with human bites for additional cases reported to have possibly been infected with HIV by human bite.

In total, 11 cases including  the case reported here were analyzed (Table 1). Three bite transmissions appeared to have been reported twice (cases 4, 8, and 9). Evidence for transmission by human bite as assessed by the respective authors varied from possible to unequivocally confirmed. Except for one case, there were no reports of other risk factors. One bitten female victim reported having had unprotected sex once with a partner of unknown HIV status after a negative HIV test prior to bite (case 2).

Eight out of 11 aggressors were reported to not having received antiretroviral therapy. In one case reported in 1996, no information on antiretroviral therapy (ART) was provided (case 4). One aggressor was on an insufficient monotherapy with zidovudine and had presented with a high viral load 11 months earlier (case 3). In this case, the victim received PEP with zidovudine. The remaining aggressor had been on highly active antiretroviral therapy (HAART) since 6 weeks prior to bite (case 10). In seven incidents, transmission of HIV-1 was reported; in the remaining 4 cases, the HIV type remained unspecified (cases 1, 2, 4, and 7). In five cases, viruses were compared by nucleic acid sequencing (cases 4, 6, 9, 10, and 11).

## Discussion

Transmission of simian immunodeficiency virus (SIV) of the chimpanzee to humans has occurred at least four times leading to HIV-1 groups M, N, O, and P. Notably, in contrast to other retroviruses, the risk of transmission of SIV by bite appears to be very low [19]. Whether SIV transmission to humans resulted from bite or other modes of transmission remains unclear.

Definite proof of HIV transmission by human bite remains difficult as reliable exclusion of other risk factors depends on truthful statements of the patients. Minimum information includes severity of the lesion, documented seroconversion of the bite victim shortly after the incident, as well as the HIV-viral load of the aggressor on the date of the bite. Nucleic acid sequencing of the viruses from the aggressor and bite victim should indicate close relatedness. However, limitations of comparative analysis of nucleic acid sequencing have to be considered, in particular after longer time periods between incident and examination.

In our opinion, at least five cases support the hypothesis that human bites may have caused HIV-1 transmission (cases 4, 6, 9, 10, and 11). In these cases, comparison of viruses from aggressors and victims showed closely related nucleic acid sequences, suggesting a common ancestor virus.

A deep bleeding wound of the victim appears to be the primary risk factor. A high viral load and bleeding oral lesions are mostly present in the aggressor (Table 1). With the exception of one case, available HIV-1 viral loads were at least 17,163 copies/ml plasma. In some reports, information on the viral load was missing, but the aggressor suffered from AIDS without receiving ART, suggesting a high viral load.

Recently, HIV-1 transmission by bite was reported from an HIV-infected person who had started HAART 6 weeks prior to the bite and had a viral load of 200 copies/ml (2.3  log_10_) 29 days thereafter (case 10). Fourteen months prior to the incident, his viral load had been 15,849 copies/ml (4.2 log_10_). This suggests that HIV-1 may also be transmitted with low viral loads if there are deep bite wounds.

Poor oral hygiene or bleeding gums associated with free blood in saliva, with HIV-containing lymphocytes and cell-free HIV, evidently increase the risk of HIV transmission by bite and have even been considered to be a precondition for transmission. Whether HIV transmission may also occur via saliva only remains speculative, but appears to be possible. In one case HIV-1 transmission appears to have occurred via saliva without contamination of blood (case 9).

Viral load in saliva parallels that in plasma, but is usually 1–2 log lower than in matched plasma samples. However, high plasma viral loads may be associated with saliva viral loads from 2.6 up to 5.9 log10 copies/ml [20]. Periodontal disease and gingival inflammation can even lead to salivary viral hyper-excretion with a fourfold or higher viral load in saliva than in plasma in individual cases [21]. Oral cavity may become an HIV-1 reservoir in individual cases.

The primary reason for the low HIV transmission risk by human bite appears to be that saliva is normally hypotonic. Hypotonicity was proven to rapidly kill infected mononuclear leukocytes, the main source of infectious HIV in the mouth. It also prevents their attachment to mucosal epithelial cells and replication of infectious HIV-1, thereby preventing transmission [22]. Cell-free HIV-1 in the mouth is inactivated by salivary inhibitors and neutralizing antibodies and usually non-infectious. However, the risk of transmission increases when saliva becomes isotonic by shed blood and when the viral load of cell-free HIV-1 in blood is high. Isotonic saliva was shown to improve the survival of infected mononuclear leucocytes, increase virus replication in the mouth, and promote cell-to-cell transmission [22].

We believe that the risk of HIV transmission by human bite should be addressed in PEP guidelines, even if the risk appears to be overall very low [2]. The number of reported cases is small, but there may have been some underreporting, particularly in high-prevalence populations of HIV infection. PEP should be recommended when a bite has led to an open skin lesion manifesting as a bleeding wound, and when the aggressor is known to be HIV-positive [2, 18]. PEP has been missed in this presented case as well as in two previously  reported bite victims (cases 9 and 10). Transmission of HIV has still a lifelong impact on our patients’ lives, while PEP has become tolerable, cost-effective, and safe nowadays [23].
